# Experimental evolution of *Saccharomyces uvarum* at high temperature yields elevation of maximal growth temperature and loss of the mitochondrial genome

**DOI:** 10.17912/micropub.biology.000831

**Published:** 2023-06-02

**Authors:** Emery R. Longan, Justin C. Fay

**Affiliations:** 1 University of Rochester, Department of Biology, Rochester, NY, 14620 USA

## Abstract

An organism’s upper thermal tolerance is a major driver of its ecology and is a complex, polygenic trait. Given the wide variance in this critical phenotype across the tree of life, it is quite striking that this trait has not proven very evolutionarily labile in experimental evolution studies of microbes. In stark contrast to recent studies, William Henry Dallinger in the 1880s reported increasing the upper thermal limit of microbes he experimentally evolved by >40°C using a very gradual temperature ramping strategy. Using a selection scheme inspired by Dallinger, we sought to increase the upper thermal limit of *Saccharomyces uvarum* . This species has a maximum growth temperature of 34-35°C, considerably lower than *S. cerevisiae* . After 136 passages on solid plates at progressively higher temperatures, we recovered a clone that can grow at 36°C, a gain of ~1.5°C. Additionally, the evolved clone lost its mitochondrial genome and cannot respire. In contrast, an induced *rho* ^0^ derivative of the ancestor shows a decrease in thermotolerance. Also, incubation of the ancestor at 34°C for 5 days increased the frequency of petite mutants drastically compared to 22°C, supporting the notion that mutation pressure rather than selection drove loss of mtDNA in the evolved clone. These results demonstrate that *S. uvarum* ’s upper thermal limit can be elevated slightly via experimental evolution and corroborate past observations in *S. cerevisiae* that high temperature selection schemes can inadvertently lead to production of the potentially undesirable respiratory incompetent phenotype in yeasts.

**Figure 1. Results of experimental evolution of Saccharomyces uvarum at high temperature:**
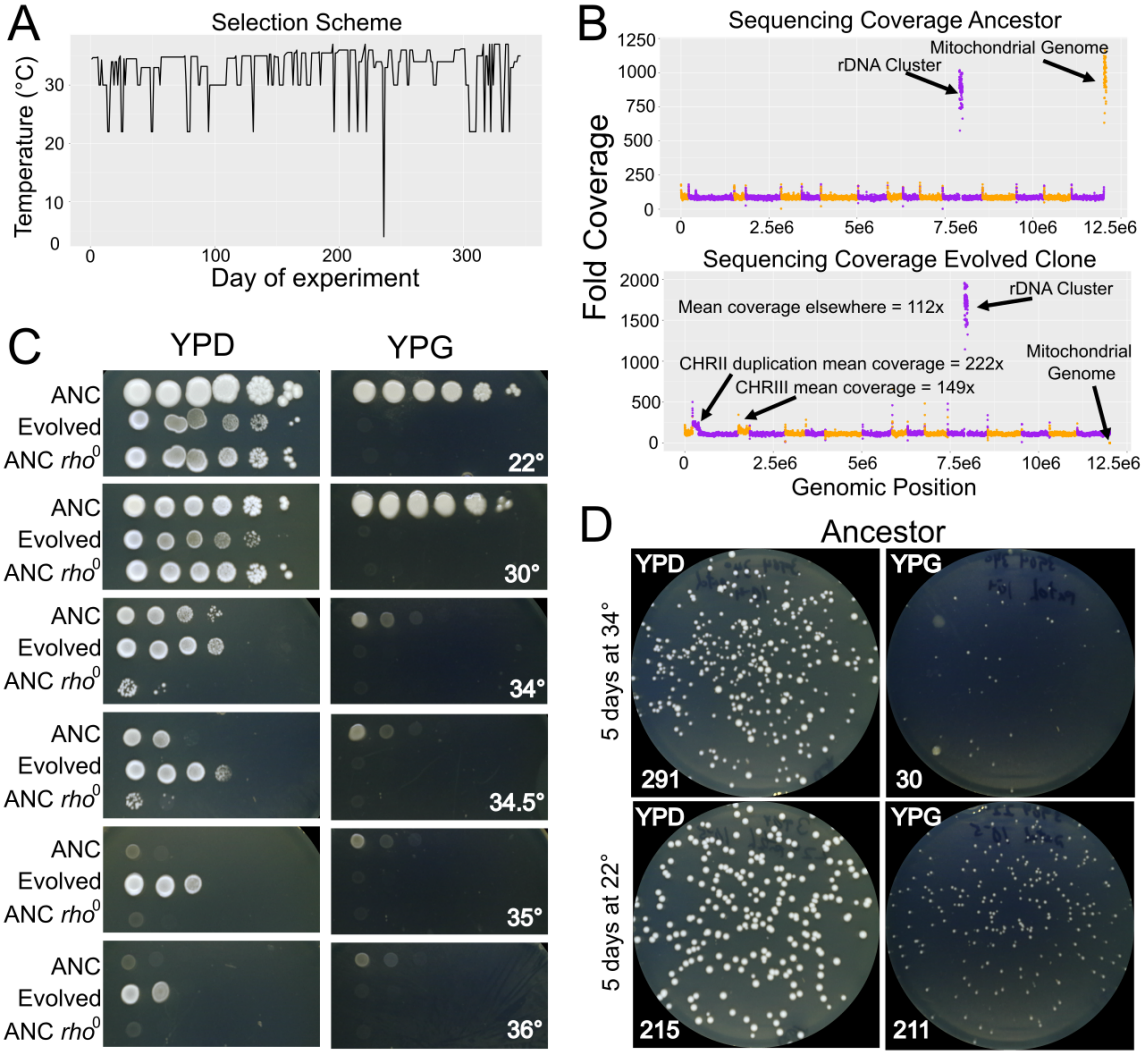
A) The selection scheme used in this study. B) Coverage plots of the whole genome sequencing data. Chromosomes are represented in numerical order by alternating colors. The evolved strain is *rho* ^0^ and has lost its mtDNA. The evolved clone also has a duplication on chromosome II (4N) and aneuploidy (3N) of chromosome III. C) Spot dilution phenotyping assays. The evolved clone has a growth defect at 22°C and 30°C relative to the ancestor (ANC) and the induced *rho* ^0^ strain (ANC *rho* ^0^ ). It also shows far more robust growth at 34.5°C and grows at 35°C and 36°C whereas the other two strains do not. The induced *rho* ^0^ strain shows a thermotolerance defect at 34°C and 34.5°C. The evolved strain and the induced *rho* ^0^ strain are incapable of respiring at any permissive temperature as expected. D) Platings for single colonies on YPD and YPG after incubation at either 22°C or 34°C for 5 days. There is a significant increase in the proportion of cells that are respiratory incompetent (incapable of producing a colony on YPG) after 34°C incubation (p<0.00001, chi-square test).

## Description

Maximal thermal tolerance is a highly polygenic phenotype that is extremely important in determining an organism’s ecology (Bennet and Lenski 1993, Rezende et al. 2014, Li et al. 2019, Abrams et al. 2021) . Across the tree of life, this phenotype varies by approximately 100°C (Stetter 1999, Moyer et al. 2017) . Despite this wide range, high temperature tolerance has proven recalcitrant to improvement by experimental evolution in studies of bacteria (Bennett and Lenski 1993, Mongold et al. 1999, Rudolph et al. 2010) , *Drosophila* (Folk et al. 2007) , and yeasts (Piotrowski et al. 2012, Caspeta et al. 2014, Satomura et al. 2016, Huang et al. 2018) . However, many of these studies used a constant selection regimen or a predetermined temperature ramping strategy. Unlike most modern studies, William Henry Dallinger used a gradual temperature ramping scheme based on his own discretion (such that the organisms were intended to be maximally stressed but not killed) to experimentally evolve a community of microbial species, and he reported drastic improvements (>40°C) in the upper thermal limit of three focal species within this community (Dallinger 1887) . In the spirit of Dallinger’s experiment, we evolved *Saccharomyces uvarum* under high temperature stress using this same type of rationale: Ensure the microbes are not killed but push the temperature as high as possible. To accomplish this, we grew *S. uvarum* as close to its upper thermal limit as possible, with occasional recovery at lower temperatures when appreciable growth halted ( Figure 1A ).

*S. uvarum* is an industrially important yeast with a thermal growth limit far lower than *S. cerevisiae* (Pulvirenti et al. 2002, Gonçalves et al. 2011). *S. uvarum* produces markedly different volatile profiles than *S. cerevisiae* in cider fermentation (Lorenzini et al. 2019) and is typically found in colder environments (de Garcia et al. 2013) . South American isolates of *S. uvarum* show the highest levels of genetic diversity, suggesting this species has only more recently spread elsewhere (Almeida et al. 2014) . Much work has been devoted to understanding the genetic basis of *S. uvarum* ’s thermal divergence from *S. cerevisiae* (Gonçalves et al. 2011, Li and Fay 2017, Li et al. 2019). Mitochondrial manipulations show that mitochondria from cryophilic yeast species confer lower thermal tolerance and the opposite for mitochondria from thermophilic species (Špírek et al. 2015, Baker et al. 2019, Li et al. 2019). To our knowledge no genetic manipulation of *S. uvarum* has conferred the capacity to grow continuously at or above 36°C other than hybridization with *S. cerevisiae* (Pulvirenti et al. 2002, Piotrowski et al. 2012, Li et al. 2019) . Despite this, *S* . *uvarum* strains that can ferment at higher temperatures presumably have practical industrial applications. Using a simple single replicate serial passaging design, we sought to use experimental evolution to raise the upper thermal limit of *S. uvarum* . This experiment lasted 346 days, included 136 transfers, and represented ~700 generations of evolution (See methods). Of note, our passaging protocol was driven by the current status of the population and the discretion of the experimenter. As such, this methodology came with the inherent drawback of pushing the upper thermal limit of only a single sample.

Following the evolution experiment, we isolated a single colony from the evolving population and performed whole genome sequencing on this clone and the ancestral strain ( Figure 1B ). We find that the evolved strain has many genetic differences compared to its ancestor. Most conspicuously, the evolved strain has a partial duplication (4N) of chromosome II and aneuploidy (3N) of chromosome III (See extended data for a list of the duplicated genes). Interestingly, chromosome III has been implicated in the evolution of thermotolerance in *S. cerevisiae* (Yona et al. 2012, Caspeta et al. 2014) , whose genome is mostly syntenic with *S. uvarum* (Scannell et al. 2011) . In addition, there are many variants present in the evolved strain that are not present in the ancestor (See extended data for the high confidence variant calls). Among the genes with disruptive variants, there were no obvious connections to thermotolerance, though there were several genes implicated in mitochondrial function (See extended data for the list of disruptive gene variants in the evolved clone).

Perhaps most interestingly, the evolved clone has lost its entire mitochondrial genome, a result that has recently been reported in high temperature experimental evolution of *S. cerevisiae* (Huang et al. 2018) . This begs the question: Is loss of mtDNA sufficient to improve the thermotolerance of *S. uvarum* ? To test this hypothesis, we generated a *rho* ^0^ derivative of the ancestral strain (Goldring et al. 1970, Stein et al. 2015) and performed spot dilution phenotyping assays ( Figure 1C ). As expected, we find that the evolved strain and the induced *rho* ^0^ strain are incapable of respiring on non-fermentable medium (YPG) at any temperature. However, we find that these strains differ markedly in their thermal growth profiles on fermentable medium (YPD). The evolved strain shows a relative defect at 22°C and 30°C compared to the induced *rho* ^0^ strain and the ancestor, but at 34°C and 34.5°C there are clear differences among all three strains ( Figure 1C ). The evolved clone has markedly increased thermotolerance as compared to the ancestor whereas the induced *rho* ^0^ strain has decreased thermotolerance compared to the ancestor. At 35°C and 36°C we see that the evolved clone can grow whereas the other two strains cannot, signifying a ~1.5°C increase in the upper thermal limit of this strain ( Figure 1C ), comparable to gains seen in prior studies using different selection schemes (Bennett and Lenski 1993, Mongold et al. 1999, Rudolph et al. 2010, Huang et al. 2018) .

These results are somewhat contradictory. If losing mtDNA decreases thermotolerance on its own, then why did this happen in our thermotolerant evolved clone? One potential explanation is that higher temperatures increase the rate at which cells lose their mtDNA, making mutation pressure the driver of this change rather than selection. To test if high temperatures induce respiratory mutants, we patched the ancestral strain on YPD and incubated the patches at either 22°C or 34°C for 5 days. Following this incubation period, we plated cells from these patches for single colonies onto YPD and YPG. The difference between the number of colony forming units on YPD and YPG should reflect the proportion of cells that are respiratory incompetent in the respective source patches. We find that there is a drastic difference in the production of respiratory incompetent cells at these two temperatures with nearly a 90% reduction in colonies recovered on YPG after 34°C incubation ( Figure 1D, p < 0.00001, chi-square test). Importantly, this experiment does not explicitly test for loss of mtDNA, but the vast majority of respiratory deficient (petite) mutants are caused by partial or complete loss of mtDNA (Sherman 1959, Chen and Clark-Walker 2000) . We also confirmed that a large fraction of the smaller colonies recovered on YPD after 34°C incubation were respiratory incompetent by patching them on YPG (See extended data). These data also corroborate similar observations in *S. cerevisiae* that long term incubation at high temperatures can yield loss of respiratory capacity and an increase in the petite mutation rate (Sherman 1956, Yčas 1956, Sherman 1958, Sherman 1959, Bulder 1964, Simões-Mendes et al. 1978, Cabeca-Silva et al. 1982, Huang et al. 2018).

Given these results, what is the broader relationship between respiratory incompetence and thermotolerance? In the context of prior studies, the answer is not obvious due to conflicting evidence pointing to loss of respiratory capacity both enhancing (Traven et al. 2001, Caspeta et al. 2014) and diminishing (Bolotin-Fukuhara et al. 1977, Zubko and Zubko 2014) thermotolerance. In our strains we see that loss of mtDNA alone reduces thermotolerance, but the evolved clone with enhanced thermotolerance is also respiratory incompetent. This raises the possibility that other mutations ameliorated the effects of mtDNA loss at high temperature. Mutations in subunits of the ATP-synthase complex, such as *ATP3* , are known to suppress the slow growth of petite strains and can increase thermotolerance (Caspeta et al. 2014, Puddu et al. 2019) . Additionally, some isolates of *S. cerevisiae* are *rho* ^0^ and show no growth defect on fermentable carbon sources (De Chiara et al. 2020) . However, we did not find mutations in subunits of the ATP-synthase complex. It is also possible that the mutations conferring thermotolerance observed in the evolved clone only have beneficial effects in respiratory deficient cells, making mtDNA loss an important step in high temperature adaptation in this experiment. Our results cannot speak to this complex scenario, but they do support a model whereby thermotolerance evolved subsequent to the loss of mtDNA.

Similar to heat stress, maintenance of the mitochondrial genome is highly polygenic and can be disrupted by a multitude of factors (Marzuki et al. 1974, Van Dyck et al. 1992, Guan 1997, Berger and Yaffe 2000, Contamine and Picard 2000) . Of these disruptive factors, increased oxidative stress is one of best characterized (Yazgan and Krebs 2012) , and heat stress is intimately tied to increases in oxidative stress via increased production of ROS (Benov and Fridovich 1992, Davidson and Schiestl 2001) . Furthermore, chemical oxidative stressors have long been known to disrupt mtDNA stability (Yazgan and Krebs 2012) . In the context of these past observations, one plausible interpretation of our results is that heat stress increases loss of mtDNA due to increased ROS. Although our data are consistent with this model, explicit investigation of this type of idea appears sparse (Del Giudice et al. 1986) .

Loss of mtDNA is also of interest because it is expected to have evolutionary consequences. These expected consequences follow from the increased nuclear mutation rate and nuclear genome instability that have been shown to accompany mtDNA loss (Kaniak-Golik and Skoneczna 2015, Tu et al. 2023) . In other experimental evolution studies, it has been widely recognized that mutator phenotypes can hitchhike to fixation due to decreased waiting time for adaptive variants (Leigh 1970, Taddei et al. 1997) , most famously in half of the lines in the Lenski lab’s long term evolution experiment (Sniegowski et al. 1997, Good et al. 2017) . It is possible that the evolved clone has accumulated more mutations due to its loss of mtDNA, but it is unlikely that there was sufficient time for hitchhiking of the *rho* ^0^ genotype with adaptive variants. At 34°C we found that only 10% of cells retain their respiratory capacity after five days. At this rate, it is doubtful there would be any respiratory positive cells remaining after a few transfers and loss of mtDNA would be inevitable. As such, the high rate and rapid loss of mtDNA also make it unlikely that this change was driven by indirect selection due to linkage disequilibrium with *de novo* adaptive variants conferring thermotolerance.

Overall, these results highlight the importance of selection schemes when trying to produce a novel phenotype. Namely, extremely strong selection can yield the inadvertent production of unintended phenotypes as well as the intended phenotype. Given that respiratory defects are often undesirable for industrial applications (Caspeta et al. 2014) , we speculate that increasing the upper thermal limit of *S. uvarum* via experimental evolution in a more useful way may require simultaneous selection against the petite phenotype. This could either be achieved via propagation on non-fermentable carbon sources such as glycerol or could possibly be done via construction of *S. uvarum* strains intolerant of mtDNA loss. Mutations in the gene *PET9* are known to cause obligate mtDNA retention in *S. cerevisiae* , but the essentiality of this gene is variable and these mutations cause a petite phenotype (Lawson et al. 1990, Giaever et al. 2002, Wang et al. 2008) . Although previous studies saw success in increasing upper thermal limit with constant or predetermined temperature ramping schemes, our study corroborates the notion that fluctuating or less rigid temperature ramping schemes can be useful alternative approaches, in addition to potentially being more ecologically relevant (Gibbs and Gefen 2009) . However, it is important to emphasize that schemes such as ours that tailor selection to the current status of the population come with the serious drawback of not being as amenable to high throughput techniques. Overall, this study demonstrates proof of principle for elevating the upper thermal limit of *S. uvarum* via experimental evolution but cautions against a lack of selection against respiratory incompetence to do so.

## Methods

*Strains*

There are two primary strains that were used in this study: YJF3904 and YJF4665. YJF3904 is a single colony isolate of YJF2602 (Li and Fay 2017) . YJF4665 is a single colony isolate of YJF3904 after 136 passages at high temperature. YJF2602 and thus YJF3904 are diploids derived from a cross of YJF1449 and YJF1450 (Scannell et al. 2011, Li et al. 2019) , which are both derivatives of the strain CBS7001. Thus, YJF3904 is a prototrophic homozygous diploid with *HO* replaced with NatMX (Goldstein and McCusker 1999) . YJF5202, used as a control for loss of mtDNA, is an ethidium bromide induced *rho* ^0^ derivative of YJF3904 (see below).

*Transfer protocol*

At each passage, cells were transferred to fresh solid medium (YPD, Bacto yeast extract 10g/L, Bacto peptone 20g/L, dextrose 20g/L). Transfers were carried out manually via patching cells from the previous patch onto fresh medium with pipette tips. Frequency of transfers and growth temperatures were chosen at the experimenter’s discretion based on how well the cells were growing. Recovery at room temperature was performed when it appeared the population was not growing appreciably, a choice similar to Dallinger’s work (Dallinger 1887) . The brief excursion to 4°C seen in Figure 1A, was a result of experimenter error .

*Generation number estimate*

We evolved *S. uvarum* over 346 days for 136 passages on solid YPD medium at high temperature. An estimate of the upper bound of the number of generations that occurred in our experiment can be inferred from mutation accumulation experiments in *S. cerevisiae* (Joseph and Hall 2004, Hall et al. 2008, Zhu et al. 2014, Liu and Zhang 2019) . The consensus of the mutation accumulation studies places the number of generations per day on solid media starting from a single cell to be ~10 generations and slightly fewer (~8.7) if the cells are stressed (Liu and Zhang 2019) . However, our cells were propagated as high-density patches, which likely conform more closely to estimates of generation number following pinning on stressful media by a Singer Rotor HDA robot (Singer Instruments). Estimates of the number of generations per pinned transfer are 4.6 for 2 days of growth and 5.3 for 3 days of growth (Miller et al. 2022) . Given this experiment had an average of 2.5 days separating transfers, this would yield an estimate of ~5 generations per transfer. Further, we can estimate a lower bound of generations on a per transfer basis as ~3 generations per transfer simply because the population was always passaged only after conspicuous growth on solid medium and was never diluted to extinction. Taken together, for this experiment we can roughly estimate that ~3000 (346*8.7) generations is the absolute upper bound (Joseph and Hall 2004, Hall et al. 2008, Zhu et al. 2014, Liu and Zhang 2019) , the lower bound is ~400 (136*3) generations, and the true number is likely ~700 (136*5) generations (Miller et al. 2022) .

*Rho* ^0^ *induction of YJF3904*

Following a protocol from Stein et al. (2015), we used ethidium bromide to induce a *rho* ^0^ derivative of YJF3904 to control for the effect of losing mtDNA on thermotolerance. Ethidium bromide has long been known to selectively inhibit mtDNA replication and leads to complete loss of mtDNA (Goldring et al. 1970) . In short, YJF3904 was inoculated into 5mls of minimal medium (yeast nitrogen base 1.7g/L, ammonium sulfate 5g/L, dextrose 20g/L) with 0.125mg of ethidium bromide and grown with shaking overnight at 25°C. The next day, the culture was diluted 1:1000 into 5mls of fresh minimal medium with 0.125mg of ethidium bromide and grown for two days at 25°C with shaking. These cells were then washed and plated on YPD for single colonies. Colonies were subsequently patched on YPD and YPG. A single isolate that had a complete respiratory defect was used in the spot dilution assays (YJF5202).

*Petite frequency assay*

YJF3904 was patched onto YPD and incubated at 22°C and 34°C respectively for 5 days. The patches were then diluted in water and plated for single colonies on YPD and YPG (Bacto yeast extract 10g/L, Bacto peptone 20g/L, glycerol 30g/L) respectively. After three days of growth at 22°C, plates were then imaged in a Phenobooth imager from Singer Instruments and colonies were counted using Phenosuite software from Singer Instruments with background subtraction set to 100. The counts on YPD and YPG were compared for the cells that had been incubated at 22°C versus 34°C using a chi-square test to determine if the proportion of cells that are petite differs between the two treatments.

*Spot dilutions*

The ancestral, evolved, and induced *rho* ^0^ strains (YJF3904, YJF4665, and YJF5202 respectively) were grown overnight at 25°C in liquid YPD with shaking. These saturated cultures were then serially diluted 1:10 five times in water and 3μl of each dilution were spotted on YPD and YPG using a multichannel pipette. After 5 days of growth, the plates were imaged using a Phenobooth imager and cropped using the Phenosuite software from Singer Instruments.

*Sequencing and variant calling*

Genomic DNA was extracted using a YeaStar genomic DNA kit (Zymo Research). Libraries were prepared using a Nextera DNA Flex Library Preparation Kit (Illumina), and sequenced as multiplexed libraries on a Hiseq X platform (150bp, paired end) via Novogene. Reads were mapped using version 0.7.17-r1188 of the Burrows-Wheeler aligner (Li 2013) to assembly ASM2755758v1 (Genbank accession no. GCA_027557585.1). Reads were marked duplicate with Picard tools version 2.12.0 and variants were called using version 4.2.6.1 of GATK using the ‘HaplotypeCaller’. Variants were subjected to a single heuristic filter, namely that only variants with different calls in the ancestor and evolved clone were retained. Variants were annotated with SNPeff (version 4.3t) using the accompanying gff file in the assembly. Raw reads produced in this study are available at NCBI BioProject PRJNA947634.

## Extended Data

Description: This file contains the image of patched colonies from the YPD plating of the patch that was incubated at 34°C for five days. . Resource Type: Image. DOI: 10.22002/yr5hv-zzv60

Description: This file contains the records of transfers and temperatures over the course of evolution.. Resource Type: Dataset. DOI: 10.22002/shdef-nkk25

Description: This file contains the list of genomic features that are duplicated in the evolved clone. . Resource Type: Dataset. DOI: 10.22002/w1aw9-64n31

Description: This is the vcf file containing all variants called in the evolved clone that are not present in the ancestor along with their annotations. . Resource Type: Dataset. DOI: 10.22002/4a6hn-ehr12

Description: This file contains a summary of the disruptive gene variants identified in the evolved clone.. Resource Type: Dataset. DOI: 10.22002/8z8pn-b1e39

## References

[R1] Abrams MB, Dubin CA, AlZaben F, Bravo J, Joubert PM, Weiss CV, Brem RB (2021). Population and comparative genetics of thermotolerance divergence between yeast species.. G3 (Bethesda).

[R2] Almeida P, Gonçalves C, Teixeira S, Libkind D, Bontrager M, Masneuf-Pomarède I, Albertin W, Durrens P, Sherman DJ, Marullo P, Hittinger CT, Gonçalves P, Sampaio JP (2014). A Gondwanan imprint on global diversity and domestication of wine and cider yeast Saccharomyces uvarum.. Nat Commun.

[R3] Baker EP, Peris D, Moriarty RV, Li XC, Fay JC, Hittinger CT (2019). Mitochondrial DNA and temperature tolerance in lager yeasts.. Sci Adv.

[R4] Bennett AF, Lenski RE (1993). EVOLUTIONARY ADAPTATION TO TEMPERATURE II. THERMAL NICHES OF EXPERIMENTAL LINES OF ESCHERICHIA COLI.. Evolution.

[R5] Benov L, Fridovich I (1995). Superoxide dismutase protects against aerobic heat shock in Escherichia coli.. J Bacteriol.

[R6] Berger KH, Yaffe MP (2000). Mitochondrial DNA inheritance in Saccharomyces cerevisiae.. Trends Microbiol.

[R7] Bolotin-Fukuhara M, Fay G, Fukuhara H (1977). Temperature-sensitive respiratory-deficient mitochondrial mutations: isolation and genetic mapping.. Mol Gen Genet.

[R8] Bulder C. J. E. A. (1964). Lethality of the petite mutation in petite negative yeasts. Antonie van Leeuwenhoek.

[R9] CabeÃ§a-Silva C., Madeira-Lopes A., Uden N. (1982). Temperature relations of ethanol-enhanced petite mutation in
*Saccharomyces cerevisiae*
: Mitochondria as targets of thermal death. FEMS Microbiology Letters.

[R10] Caspeta L, Chen Y, Ghiaci P, Feizi A, Buskov S, Hallström BM, Petranovic D, Nielsen J (2014). Biofuels. Altered sterol composition renders yeast thermotolerant.. Science.

[R11] Chen XJ, Clark-Walker GD (2000). The petite mutation in yeasts: 50 years on.. Int Rev Cytol.

[R12] Contamine V, Picard M (2000). Maintenance and integrity of the mitochondrial genome: a plethora of nuclear genes in the budding yeast.. Microbiol Mol Biol Rev.

[R13] Dallinger, William H. 1887. The president's address. Journal of the Royal Microscopical Society 7.2: 185-199. (retrievable at: https://ia800708.us.archive.org/view_archive.php?archive=/22/items/crossref-pre-1909-scholarly-works/10.1111%252Fj.1365-2818.1882.tb01547.x.zip&file=10.1111%252Fj.1365-2818.1887.tb01566.x.pdf)

[R14] Davidson JF, Schiestl RH (2001). Mitochondrial respiratory electron carriers are involved in oxidative stress during heat stress in Saccharomyces cerevisiae.. Mol Cell Biol.

[R15] De Chiara M, Friedrich A, Barré B, Breitenbach M, Schacherer J, Liti G (2020). Discordant evolution of mitochondrial and nuclear yeast genomes at population level.. BMC Biol.

[R16] Del Giudice L., Massardo D. R., Manna F., Wolf K. (1986). Isolation and characterization of a conditional mutant in Saccharomyces cerevisiae producing rho° petites at the non-permissive temperature. Current Genetics.

[R17] Folk DG, Hoekstra LA, Gilchrist GW (2007). Critical thermal maxima in knockdown-selected Drosophila: are thermal endpoints correlated?. J Exp Biol.

[R18] de Garcia Virginia, Libkind Diego, Moliné Martín, Rosa Carlos A., Giraudo Maria Rosa (2013). Cold-Adapted Yeasts in Patagonian Habitats. Cold-adapted Yeasts.

[R19] Giaever G, Chu AM, Ni L, Connelly C, Riles L, Véronneau S, Dow S, Lucau-Danila A, Anderson K, André B, Arkin AP, Astromoff A, El-Bakkoury M, Bangham R, Benito R, Brachat S, Campanaro S, Curtiss M, Davis K, Deutschbauer A, Entian KD, Flaherty P, Foury F, Garfinkel DJ, Gerstein M, Gotte D, Güldener U, Hegemann JH, Hempel S, Herman Z, Jaramillo DF, Kelly DE, Kelly SL, Kötter P, LaBonte D, Lamb DC, Lan N, Liang H, Liao H, Liu L, Luo C, Lussier M, Mao R, Menard P, Ooi SL, Revuelta JL, Roberts CJ, Rose M, Ross-Macdonald P, Scherens B, Schimmack G, Shafer B, Shoemaker DD, Sookhai-Mahadeo S, Storms RK, Strathern JN, Valle G, Voet M, Volckaert G, Wang CY, Ward TR, Wilhelmy J, Winzeler EA, Yang Y, Yen G, Youngman E, Yu K, Bussey H, Boeke JD, Snyder M, Philippsen P, Davis RW, Johnston M (2002). Functional profiling of the Saccharomyces cerevisiae genome.. Nature.

[R20] Gibbs AG, Gefen E. Physiological adaptation in laboratory environments. Experimental evolution. Univ. of California Press, Berkeley (2009).

[R21] Goldring ES, Grossman LI, Krupnick D, Cryer DR, Marmur J (1970). The petite mutation in yeast. Loss of mitochondrial deoxyribonucleic acid during induction of petites with ethidium bromide.. J Mol Biol.

[R22] Goldstein AL, McCusker JH (1999). Three new dominant drug resistance cassettes for gene disruption in Saccharomyces cerevisiae.. Yeast.

[R23] Gonçalves P, Valério E, Correia C, de Almeida JM, Sampaio JP (2011). Evidence for divergent evolution of growth temperature preference in sympatric Saccharomyces species.. PLoS One.

[R24] Good BH, McDonald MJ, Barrick JE, Lenski RE, Desai MM (2017). The dynamics of molecular evolution over 60,000 generations.. Nature.

[R25] Guan MX (1997). Cytoplasmic tyrosyl-tRNA synthetase rescues the defect in mitochondrial genome maintenance caused by the nuclear mutation mgm104-1 in the yeast Saccharomyces cerevisiae.. Mol Gen Genet.

[R26] Hall DW, Mahmoudizad R, Hurd AW, Joseph SB (2008). Spontaneous mutations in diploid Saccharomyces cerevisiae: another thousand cell generations.. Genet Res (Camb).

[R27] Huang CJ, Lu MY, Chang YW, Li WH (2018). Experimental Evolution of Yeast for High-Temperature Tolerance.. Mol Biol Evol.

[R28] Joseph SB, Hall DW (2004). Spontaneous mutations in diploid Saccharomyces cerevisiae: more beneficial than expected.. Genetics.

[R29] Kaniak-Golik A, Skoneczna A (2015). Mitochondria-nucleus network for genome stability.. Free Radic Biol Med.

[R30] Lawson JE, Gawaz M, Klingenberg M, Douglas MG (1990). Structure-function studies of adenine nucleotide transport in mitochondria. I. Construction and genetic analysis of yeast mutants encoding the ADP/ATP carrier protein of mitochondria.. J Biol Chem.

[R31] Leigh, Egbert Giles (1970). Natural Selection and Mutability. The American Naturalist.

[R32] Li Heng (2013). Aligning sequence reads, clone sequences and assembly contigs with BWA-MEM.

[R33] Li XC, Fay JC (2017). Cis-Regulatory Divergence in Gene Expression between Two Thermally Divergent Yeast Species.. Genome Biol Evol.

[R34] Li XC, Peris D, Hittinger CT, Sia EA, Fay JC (2019). Mitochondria-encoded genes contribute to evolution of heat and cold tolerance in yeast.. Sci Adv.

[R35] Liu H, Zhang J (2019). Yeast Spontaneous Mutation Rate and Spectrum Vary with Environment.. Curr Biol.

[R36] Lorenzini Marilinda, Simonato Barbara, Slaghenaufi Davide, Ugliano Maurizio, Zapparoli Giacomo (2019). Assessment of yeasts for apple juice fermentation and production of cider volatile compounds. LWT.

[R37] Marzuki S, Hall RM, Linnane AW (1974). Induction of respiratory incompetent mutants by unsaturated fatty acid depletion in Saccharomyces cerevisiae.. Biochem Biophys Res Commun.

[R38] Miller JH, Fasanello VJ, Liu P, Longan ER, Botero CA, Fay JC (2022). Using colony size to measure fitness in Saccharomyces cerevisiae.. PLoS One.

[R39] Mongold JA, Bennett AF, Lenski RE (1999). EVOLUTIONARY ADAPTATION TO TEMPERATURE. VII. EXTENSION OF THE UPPER THERMAL LIMIT OF ESCHERICHIA COLI.. Evolution.

[R40] Moyer Craig L., Eric Collins R., Morita Richard Y. (2017). Psychrophiles and Psychrotrophs. Reference Module in Life Sciences.

[R41] Piotrowski JS, Nagarajan S, Kroll E, Stanbery A, Chiotti KE, Kruckeberg AL, Dunn B, Sherlock G, Rosenzweig F (2012). Different selective pressures lead to different genomic outcomes as newly-formed hybrid yeasts evolve.. BMC Evol Biol.

[R42] Puddu F, Herzog M, Selivanova A, Wang S, Zhu J, Klein-Lavi S, Gordon M, Meirman R, Millan-Zambrano G, Ayestaran I, Salguero I, Sharan R, Li R, Kupiec M, Jackson SP (2019). Genome architecture and stability in the Saccharomyces cerevisiae knockout collection.. Nature.

[R43] Pulvirenti, A., Zambonelli, C., Todaro, A., & Giudici, P. (2002). Interspecific hybridisation by digestive tract of invertebrates as a source of environmental biodiversity within the *Saccharomyces cerevisiae* . Annals of microbiology, *52* (3):245-256.

[R44] Rezende Enrico L., Castañeda Luis E., Santos Mauro (2014). Tolerance landscapes in thermal ecology. Functional Ecology.

[R45] Rudolph B, Gebendorfer KM, Buchner J, Winter J (2010). Evolution of Escherichia coli for growth at high temperatures.. J Biol Chem.

[R46] Satomura A, Miura N, Kuroda K, Ueda M (2016). Reconstruction of thermotolerant yeast by one-point mutation identified through whole-genome analyses of adaptively-evolved strains.. Sci Rep.

[R47] Scannell DR, Zill OA, Rokas A, Payen C, Dunham MJ, Eisen MB, Rine J, Johnston M, Hittinger CT (2011). The Awesome Power of Yeast Evolutionary Genetics: New Genome Sequences and Strain Resources for the Saccharomyces sensu stricto Genus.. G3 (Bethesda).

[R48] Simões-Mendes B, Madeira-Lopes A, van Uden N (1978). Kinetics of petite mutation and thermal death in Saccharomyces cerevisiae growing at superoptimal temperatures.. Z Allg Mikrobiol.

[R49] SHERMAN F (1956). The heat inactivation and production of cytochrome deficiency in yeast.. Exp Cell Res.

[R50] Sherman F (1958). A STUDY OF THE EFFECTS OF ELEVATED TEMPERATURES ON THE GROWTH AND INHERITANCE OF SACCHAROMYCES CEREVISIAE (thesis).

[R51] SHERMAN F (1959). The effects of elevated temperatures on yeast. II. Induction of respiratory-deficient mutants.. J Cell Comp Physiol.

[R52] Sniegowski PD, Gerrish PJ, Lenski RE (1997). Evolution of high mutation rates in experimental populations of E. coli.. Nature.

[R53] Špírek M, Poláková S, Jatzová K, Sulo P (2015). Post-zygotic sterility and cytonuclear compatibility limits in S. cerevisiae xenomitochondrial cybrids.. Front Genet.

[R54] Stein A, Kalifa L, Sia EA (2015). Members of the RAD52 Epistasis Group Contribute to Mitochondrial Homologous Recombination and Double-Strand Break Repair in Saccharomyces cerevisiae.. PLoS Genet.

[R55] Stetter KO (1999). Extremophiles and their adaptation to hot environments.. FEBS Lett.

[R56] Taddei F, Radman M, Maynard-Smith J, Toupance B, Gouyon PH, Godelle B (1997). Role of mutator alleles in adaptive evolution.. Nature.

[R57] Traven A, Wong JM, Xu D, Sopta M, Ingles CJ (2000). Interorganellar communication. Altered nuclear gene expression profiles in a yeast mitochondrial dna mutant.. J Biol Chem.

[R58] Tu Xinyu, Wang Fan, Liti Gianni, Breitenbach Michael, Yue Jia-Xing, Li Jing (2023). Spontaneous Mutation Rates and Spectra of Respiratory-Deficient Yeast. Biomolecules.

[R59] Van Dyck E, Foury F, Stillman B, Brill SJ (1992). A single-stranded DNA binding protein required for mitochondrial DNA replication in S. cerevisiae is homologous to E. coli SSB.. EMBO J.

[R60] Wang X, Salinas K, Zuo X, Kucejova B, Chen XJ (2008). Dominant membrane uncoupling by mutant adenine nucleotide translocase in mitochondrial diseases.. Hum Mol Genet.

[R61] Yazgan O, Krebs JE (2012). Mitochondrial and nuclear genomic integrity after oxidative damage in Saccharomyces cerevisiae.. Front Biosci (Landmark Ed).

[R62] Yčas M. 1956. A hereditary cytochrome deficiency appearing in yeast grown at an elevated temperature. Exp. Cell Res. 10: 746.

[R63] Yona AH, Manor YS, Herbst RH, Romano GH, Mitchell A, Kupiec M, Pilpel Y, Dahan O (2012). Chromosomal duplication is a transient evolutionary solution to stress.. Proc Natl Acad Sci U S A.

[R64] Zhu YO, Siegal ML, Hall DW, Petrov DA (2014). Precise estimates of mutation rate and spectrum in yeast.. Proc Natl Acad Sci U S A.

[R65] Zubko EI, Zubko MK (2013). Deficiencies in mitochondrial DNA compromise the survival of yeast cells at critically high temperatures.. Microbiol Res.

